# Finding cancer's weakest link

**DOI:** 10.18632/oncotarget.396

**Published:** 2011-12-22

**Authors:** Nicole M. Sodir, Gerard I. Evan

**Affiliations:** ^1^ Department of Pathology and Helen Diller Family Comprehensive Cancer Center, University of California San Francisco, CA 94143, USA; ^2^ Department of Biochemistry, University of Cambridge, Cambridge CB2 1GA, UK

**Keywords:** Myc inhibition, tumor, microenvironment, cancer therapeutics

## Abstract

The biological programs of vertebrates exhibit a remarkable degree of functional degeneracy, adaptive compensation and robustness, to preserve homeostasis and generate reproducible phenotypic outputs irrespective of variations in signal strength, noise and quality. Cancers are difficult to treat not only because they are so mechanistically diverse but also because they adapt or evolve in response to any pharmacological elective pressure we impose upon them. Hence, an ideal cancer drug target would exert a function both necessary for cancer cell survival and functionally non-redundant, rendering it impossible for tumor cells to compensate for, or evolve independence from, the inhibitory effect of any drug aimed at that target. In this review, we discuss the unique, non-degenerate and highly pleiotropic role played by Myc in coordinating, engaging and maintaining the diverse intracellular and extracellular programs required for cell proliferation in vivo. These properties make Myc a compelling candidate cancer drug target, at least in principle: an assertion recently reinforced by new *in vivo* genetic data.

## WHY ARE CANCERS DIFFICULT TO CURE?

Cancers are extremely multifarious diseases that arise through the accumulation in somatic cells of mutations in the genes that regulate and restrain cell multiplication, survival, repair, movement and invasion. The process is “Darwinian” – oncogenic mutations occur at random and fate of the mutant cells and their progeny is determined by their relative “fitness” – in this case, their relative capacities to survive and propagate in and, eventually, spread from, their requisite somatic niche. While the mutations that fuel tumor evolution occur randomly, their evolutionary tradjectories are shaped by the selective pressures that normally limit untoward somatic cell growth – principally the restricted and tightly regulated availability of mitogenic and survival signals, barriers to cell migration and macroscopic tumor expansion, and the relentless vigilance of innate tumor suppressor pathways. Not surprisingly, given the haphazard and aleatory way in which individual cancers evolve, each cancer in each patient is unique. Indeed, it is now clear that there is a substantial genetic diversity even within tumor cell populations in each individual tumor. Such innate genetic diversity feeds the engines of further evolution – in essence, each tumor must be treated as an evolving species rather than as a unitary object.

Many point to this alarming genetic diversity in cancers – an inevitable consequence of the haphazard and directionless way that each cancer evolves, as the principal reason why cancers have proven so difficult to treat. The idea is that past cancer therapies were applied fairly indiscriminately against many different genetic pathologies and what is needed is a therapy that is tailored to the particular complement of genetic lesions in each individual's cancer – cancer therapy will need to be personalized. With the advent of new-targeted drugs, engineered to inhibit specific defects in specific cancers types, many hope that this may, at least, be feasible. However, this disregards a more fundamental reason for intractability of cancers, which is that cancer cells adapt and evolve. Biological “wetwear” is inherently noisy and protean and works reliably only because it has evolved to be robust. Such robustness involves a great deal of functional redundancy, intrinsic error correction and inherent self-organization. Targeting a biological system with a drug is just another source of noise and variability and, if it is able, the system spontaneously compensates, re-routing signals and restoring homeostasis. Then, in those rare situations where compensation is insufficient, evolution takes over: spontaneously arising mutant cancer clones resistant to the targeted drug rapidly outgrow their incapacitated siblings and the patient relapses. The chilling truth is that it doesn't matter how effective/specific a therapy is – if its target function can be circumvented by compensation or evolution, that therapy will inevitably fail – clinically, it is just a matter of when.

## ROBUSTNESS AND SWITCHABILITY IN TUMORS AND THEIR MICROENVIRONMENT

Can anything be done to gainsay two such formidable adversaries as compensation and adaptation? One appealing strategy has been to limit the capacity for evolution of resistance by targeting the genetically normal and stable stromal compartment of tumors rather than genetically unstable cancer cells themselves. Expansion and maintenance of macroscopic tumors are dependent on their somatic microenvironment, a complicated melange of stromal, vascular and inflammatory cell types that provides necessary oxygen, nutrients, and survival factors. This obligate role played by the genetically stable tumor microenvironment in macroscopic tumor maintenance has spurred a variety of novel therapeutic approaches aimed at neutralizing tumor cell-nonautonomous components of the tumor mass, such as tumor vasculature and inflammation. Unfortunately, the effectiveness of such therapies is thwarted by the highly robust and functionally degenerate nature of the extracellular programmes that maintain the tumor microenvironment, making them highly adaptive - inhibition of one node triggers homeostatic compensatory re-routing through others [1-4]. Such functional redundancy, together with the highly interdependent and reciprocal relationship between tumor cells and their microenvironment, involving interactions with diverse stromal, vascular and inflammatory cell types, makes it difficult to dissect out the cause-and-effect dependencies linking the two and, consequently, where best to intervene for optimal therapeutic benefit.

An alternative potential strategy for thwarting cancer cell compensation and evolution is to identify therapeutic targets that are essential for the survival of cancer cells but are not functionally redundant and whose inhibition, therefore, cannot be circumvented by compensation or evolution. Do such targets exist? Can we make drugs that inhibit them? How bad might the side effects of such therapies be, given that such central engines of biology are likely to serve important functions in many normal cells and tissues? These become the critical questions.

## MYC AS A FUNCTIONALLY NON-REDUNDANT NODE IN GROWTH SIGNALING

**Figure 1:**
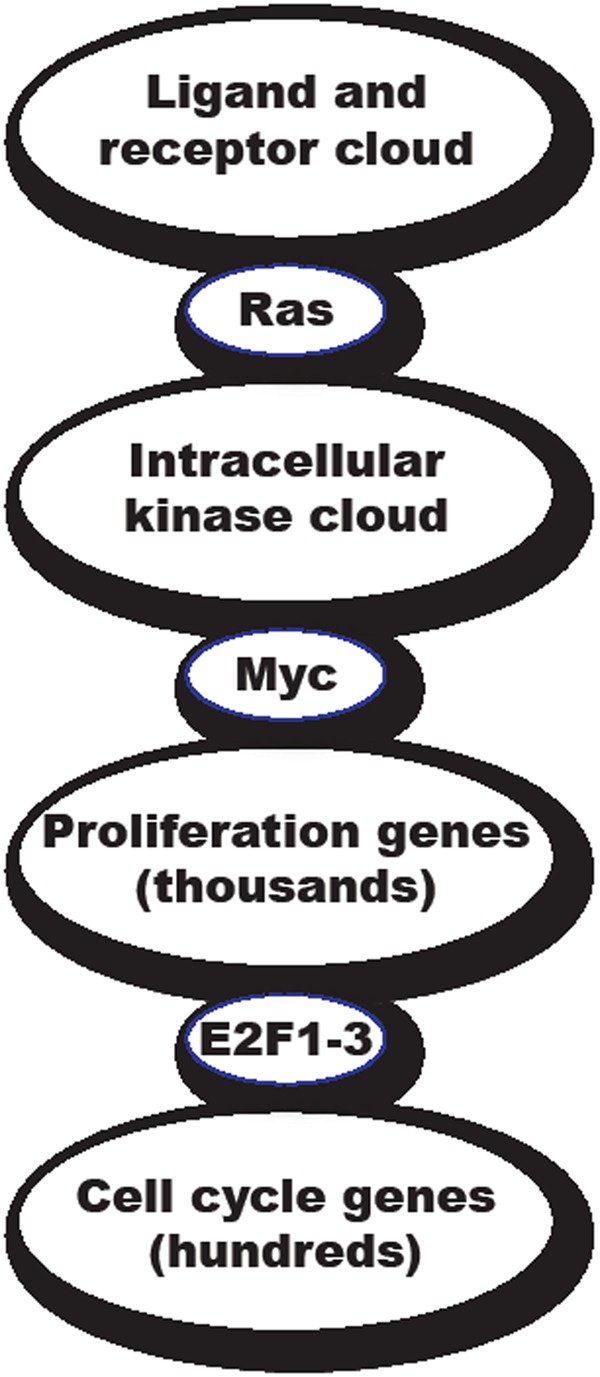
Schematic model of how the need for robustness in biological systems is reconciled with the need for binary switchability The consolidated outputs from robust, self-correcting and functionally degenerate information-gathering and information-processing “clouds” (e.g. receptor tyrosine kinases, intracellular kinases, disparate coordinated programs mediating somatic cell replication and propagation) are hypothesized to funnel down into functionally non-degenerate, go/no go switches such Ras, Myc and the activating E2F (E2F1, 2 & 3a) proteins. The obligate and functionally non-degenerate properties of Myc, Ras and E2F make them a unique class of therapeutic drug target, whose inhibition cannot easily be circumvented by compensatory or evolutionary mechanisms. However, the very essentialness of these targets raises the specter of severe side effects. In this regard, Myc has the advantage that, since its sole biological role appears to be in cell proliferation, the only side effects of Myc inhibition are likely to affect regenerating tissues.

The robustness of biological programs is a prerequisite for self-organizing homeostasis and for the generation of reproducible phenotypic outputs even when the signals directing such outputs are variable, noisy and capricious. Such robustness is manifest in the remarkable capacity of biological systems to resist and correct for perturbation and to rebuild after damage. Nonetheless, biological systems must also have the capacity to drive binary decisions – cells either arrest or proliferate, live or die, remain a progenitor or differentiate, stay put or move. How can the capacity to execute unequivocal decisions be accommodated with a biology that has evolved to resist perturbation? We suggest that these two antithetical imperatives are reconciled by connecting functionally degenerate, robust networks with functionally non-degenerate network nodes. In this way, the net outputs of diffuse, robust networks are distilled into binary Go/No-go decisions. This idea is depicted for cell growth signaling in Figure 1: the consolidated outputs from highly degenerate information gathering and information processing effector “clouds” (e.g. ligated receptor tyrosine kinases, intracellular kinases, proliferation genes) are funneled into functionally non-degenerate nodes such as Ras, Myc and mitogenic E2F (E2F1, 2 & 3a), whose activities serve to commit information flow down to the next functionally robust and degenerate cloud of effectors. When it comes to treating such a protean and evolutionarily adaptable pathology as cancer, such ubiquitously essential and functionally non-redundant nodes are especially intriguing pharmacological targets. In this review, we consider the potential of inhibiting Myc as a general treatment for cancers.

Myc is a basic helix–loop–helix leucine zipper transcription factor that coordinates the diverse transcriptional programs necessary for cell growth, proliferation, invasion, expansion and angiogenesis, as well as a variety of protective checkpoint mechanisms such as growth arrest and apoptosis [5-7]. Myc's highly pleiotropic effects are mirrored by its enormous range of gene targets. Expression array, SAGE, chromatin IP, promoter scanning and whole cell proteomic approaches indicate thousands of Myc target genes with roles in virtually every aspect of cell and tissue behavior [8-17]. Myc is present at very low levels in normal cells; both the short-lived c-Myc protein and its equally short-lived mRNA are tightly and continuously dependent upon external mitogens.

Because of the unique and pivotal role that only Myc can fill in coordinating the transcription of its thousands of target genes, Myc is functionally non-redundant. And while there are three discrete members of the Myc transcription factor family (of which at least two, c-Myc and N-Myc, appear largely isofunctional), most adult cells rely solely on c-Myc to integrate the transcription of their proliferative programs. By contrast, N-Myc and L-Myc are restricted to various stages of tissue development, although recently it has become evident that N-Myc, and perhaps L-Myc, are expressed in stem and progenitor compartments of both normal and neoplastic adult tissues. c-Myc null mice fail to develop beyond embryonic day 9.5 [18] while Rat1 fibroblasts (which, incidentally, express neither N-myc or L-myc) in which both copies of the c-*myc* gene have been ablated by targeted homologous recombination exhibit greatly reduced rates of RNA, protein translation and protein degradation and profoundly slowed proliferation, with severe defects at multiple points in the cell cycle [19]. These observations indicate that, in effect, Myc serves as the unique, functionally non-redundant node that relays diverse upstream intracellular mitogenic signals to the legion of downstream genetic programs that implement cell proliferation (Figure 1).

Intriguingly, Myc is deregulated and/or overexpressed in the majority of cancers, where it hijacks the same diverse intracellular and extracellular regenerative programs that drive normal somatic cell expansion. Ectopic activation of c-Myc alone is usually insufficient to drive tumorigenesis without the cooperation of additional sporadic oncogenic lesions, in great part because elevated Myc expression is a potent trigger of apoptosis [20-25], one of several intrinsic tumor suppressive mechanisms that antagonizes the neoplastic potential of dominant oncogenes [26]. It may be for this reason that oncogenic activation of Myc is infrequently a driving lesion in cancers, at least in early stage tumors. Rather, Myc expression is typically deregulated and elevated due to oncogenic mutations in upstream signaling pathways that relentlessly and inaptly drive endogenous Myc expression.

The *in vivo* oncogenic impact of deregulated Myc has been explored in detail using a variety of switchable transgenic mouse models in which regulatable forms of Myc, expressed at high level, have been targeted to specific tissues. Using a conditional form of Myc fused to a modified hormone binding domain of the estrogen receptor (MycER^TAM^) that is responsive only to the synthetic steroid 4-hydroxytamoxifen (4-OHT) [27], activation of Myc alone was shown to trigger rapid proliferation and disruption of differentiation when targeted to suprabasal keratinocytes *in vivo*, resulting in dramatic papillomatosis that rapidly regressed upon subsequent inactivation of MycER^TAM^ [28]. Intriguingly, sustained Myc activation was, alone, sufficient to induce not only epidermal cell expansion but also the rapid and dramatic onset of dermal angiogenesis in regions adjacent to the Myc-driven papillomas in great part, due to the elevated levels of VEGF secreted by Myc-expressing keratinocytes. Such angiogenesis is continuously dependent on Myc activity since subsequent Myc de-activation triggers abrupt vascular collapse and regression of papillomas [28]. In a different switchable Myc transgenic model, acute activation of MycER^TAM^ in pancreatic β cells *in vivo* triggered rapid and wholesale β cell proliferation: in this case, however, β cell expansion was curtailed by concomitant induction of β cell apoptosis, resulting in net islet attrition. Nonetheless, when such apoptosis was blocked by co-expression of the apoptosis inhibitor Bcl-x_L_, Myc activation triggered sustained and progressive β cell expansion, rapidly leading to the formation of large, locally invasive, inflammatory and highly angiogenic β cell tumors [29]. The rapidity and synchrony with which such Myc-driven β cell neoplasms acquire such ostensibly diverse neoplastic attributes indicates that all such traits are directly instructed by Myc, and not the result of subsequent mutations [29]. Moreover, just as in skin, subsequent c-Myc deactivation triggered rapid and complete regression of islet tumors, accompanied by collapse of tumor vasculature and microenvironment. Several similar studies in other tissues confirm that, in most cases, sustained Myc expression is required to maintain Myc-driven tumors [30-33], although there are occasional exceptions to this due to compensatory activation of alternate oncogenic signaling pathways [34]. In such instances, Myc de-induction is accompanied by growth arrest, terminal differentiation and vascular collapse. Indeed, even brief inactivation of Myc appears to offer potential therapeutic value [35-37], fueling the contemporary concept of “oncogene addiction” – an hypothesized acquired dependency in which tumor cells become dependent on the aberrantly sustained flux running through the oncogenic lesions. Given the capacity of Myc to drive and maintain both angiogenesis and local inflammation and stromal remodeling, at least some of this dependency appears to be due to collapse of the tumor microenvironment when oncogenic Myc is turned off [38].

Indeed, a detailed kinetic expression array analysis following acute activation and subsequent deactivation of Myc in pancreatic β cells revealed a clear and direct instructive role for Myc in initiating and maintaining the sustaining interplay between tumor cells and their microenvironment [39]. Acute activation of c-Myc was found to trigger expression and release of interleukin 1β (IL-1β), a pleiotropic cytokine implicated in acute and chronic inflammation and a potent inducer of metalloproteinase activity. This, in turn, triggered the release of pre-existing, extracellular matrix-bound VEGF, liberating it to bind its cognate receptor on endothelial cells, and thereby flicking the angiogenic switch [40]. c-Myc activation also triggers the rapid induction of a cluster of chemokines that likely mediate recruitment to the tumor site of various inflammatory cells, including mast cells, macrophages and neutrophils [39]. A subsequent study demonstrated that mast cell recruitment is essential for macroscopic tumor expansion and maintenance of tumor vasculature [41]. Thereafter, a mutually supportive, reciprocal interaction develops between the Myc-driven β tumor cells that is necessary for growth and maintenance of tumors and requires continuous Myc activity for its maintenance.

## MYC AS A TARGET FOR CANCER THERAPY

Such studies indicate the pivotal role that oncogenic Myc plays in driving and maintaining multiple aspects of the tumor environment, including angiogenesis, stromal remodeling, tumor invasion, and recruitment of inflammatory pathways. However, as already pointed out, the majority of human tumors are not driven by Myc mutations but by “upstream” activated oncogenes mutations in most cancers lie “upstream” in RTKs, Ras, Wnt or a host of other signaling pathways. In such tumors, the role played by endogenous Myc in tumor induction, progression and maintenance has, until recently, remained unclear. A study by Baudino *et al*. indicated a more general, physiological role for Myc in angiogenesis - *c-Myc–/–* embryos exhibit deficits in vasculogenesis, angiogenesis and primitive erythropoiesis and have reduced expression of VEGF and the angiogenic factor angiopoietin-2 as well as elevated levels of the angiogenic inhibitors thrombospondin-1 and angiopoietin-1 in murine embryonic stem cells and yolk sacs [42]. More recently, the role played by *endogenous* Myc, as opposed to ectopically activated oncogenic Myc, in the maintenance of cancers driven by other oncogenic mechanisms, addressed the extent to which endogenous Myc acts as a common downstream conduit of the upstream oncogenic signals that drive most human cancers. To do this, a dominant interfering mutant of Myc, Omomyc, was employed that displaces Myc's obligate endogenous partner Max and thereby prevents Myc from transactivating its target genes via its concensus E-box elements [43-45]. By placing systemic Omomyc expression under the control of doxycyline, Myc function may be reversibly blocked globally in all tissues *in vivo*. Systemic inhibition of Myc in both the well-established *LSL–Kras^G12D^* murine model of non-small cell lung cancer [46] and the *RIP1-Tag2* model of pancreatic insulinomas driven by SV40 T/t antigens [47] triggered rapid and wholesale regression of incipient and established tumors [48] [49], confirming that endogenous Myc function is required for maintenance of tumors driven by diverse oncogenic mechanisms. A careful kinetic analysis of Myc-inhibition-induced tumor regression in the *RIP1-Tag2* model demonstrated that inhibition of endogenous Myc triggers the rapid collapse of the tumor microenvironment, with concomitant apoptosis of endothelial cells, suppression of all detectable interaction between VEGF and its receptor, inhibition of both recruitment and/or retention of the inflammatory cells macrophages and neutrophils, vascular collapse and hypoxia [49, 50]. Moreover, such collapse of the tumor microenvironment temporally preceeds death of tumor cells or detectable regression of β cell tumor masses, strongly suggesting that collapse of the tumor microenvironment is a cause, not a consequence, of tumor regression. Furthermore, regression of *RIP1-Tag2* tumors occurs with identical kinetics and gross pathology when endogenous Myc is inhibited solely in the β cell compartment, confirming that it is endogenous Myc, within the tumor cells themselves, that is responsible for establishing and maintaining the signal cross-talk between tumor and microenvironment [49].

As discussed, the ideal cancer drug target must fulfill an essential function that is continuously required for tumor maintenance but dispensable (at least in the short term) for maintenance and function of any normal tissues. For optimal therapeutic efficacy, the target should also be functionally non-redundant – in that way, its inhibition cannot be circumvented by adaptation, compensation or evolution. Finding such targets, should they exist, remains the most pressing problem in contemporary translational cancer research. In many ways Myc looks like it fulfills all the above criteria: it acts as a non-redundant downstream node through which all upstream oncogenic and mitogenic signals pass, relaying those signals on the diverse genes that implement the complex and coordinated process of cell proliferation. It is essential for effective cell proliferation and, because its actions appear broadly limited to cell proliferation, the spectrum of side effects caused by its systemic inhibition is likely to be limited only to proliferating tissues. Unfortunately, several factors continue to diminish enthusiasm for pharmacological targeting of Myc. First, Myc exerts its manifold effects mostly through protein-protein interactions, an unfashionable and often difficult class of biological process to perturb with small molecule drugs, although recent advances in tethering and macrolide chemistries now make this more feasible. Second, aberrant Myc expression in most human cancers is usually not due to mutation in the Myc gene itself but a consequence of its induction by ‘upstream’ oncogenic signals. The therapeutic utility of inhibiting Myc when its aberrant expression is a consequence, not a cause, of oncogenesis is unclear. Third, Myc is essential for proliferation and stem cell compartment maintenance of regenerative adult tissues such as the gastrointestinal tract, skin and bone marrow. Hence, blocking Myc function systemically might trigger unacceptably severe side effects, suppressing proliferation in those same tissues that are vulnerable to classical chemo- and radiotherapy. Finally, if Myc is required solely for cell proliferation, at best its inhibition is likely to cause tumor cell arrest, not death, and as such would have limited therapeutic utility. Together, such concerns have greatly undermined the credibility of Myc inhibition as an anti-cancer strategy.

Surprisingly, however, the genetic studies in mice in which Myc function is systemically inhibited [48] offer us a great deal of reassurance. Although systemic Myc inhibition induces profound growth suppression in adult proliferating tissues – effectively stalling proliferation in regenerative tissues such as intestine, bone marrow, skin and testis – it is remarkably well tolerated for extended periods, in great part because Myc inhibition does not elicit any disruption of tissue integrity. Mice exhibit no signs of distress and maintain their weight and normal blood chemistry. Moreover, the side effects of systemic myc inhibition on all normal tissues are completely reversible upon restoration of endogenous Myc function [48]. Yet more surprisingly, given its lack of toxicity in normal proliferating tissues, Myc inhibition has an unexpectedly potent cytotoxic impact on tumor cells, triggering rapid and complete regression of both K-Ras^*G12D*^-driven non-small cell lung cancer [48] and of SV40 large T/small t antigen-driven pancreatic islet tumors in *RIP1-Tag2* mice [49], irrespective of stage of the tumor progression. At least part of the basis for this unanticipated tumor specificity is that sustained Myc activity is required by tumor cells to maintain the continuous output of angiogenic and pro-inflammatory factors needed to maintain the peculiarly factor-dependent microenvironment needed to support solid tumors: hence, Myc inhibition rapidly triggers the collapse of the tumor microenvironment. Of note, out of literally hundreds of individual lung tumors in the KRas^*G12D*^-driven NSCLC mouse model, no tumors resistant to Myc inhibition have ever emerged (Soucek & Evan, unpublished data), consistent with the unique, essential non-redundant role that Myc plays: tumor cells cannot circumvent their need for Myc by compensation or evolution. It is also, perhaps, telling that, out of several hundred individual β-cell *RIP1-Tag2* tumors, each comprised of many thousands of tumor cells in which both Rb and p53 had been simultaneously incapacitated, the only occasional tumors that emerged as resistant to doxycycline had all lost expression of the omomyc transgene [49]. Hence, the only mechanism by which tumors can circumvent Omomyc inhibition of Myc is to “break” the model system. This is an exception that seems to prove the rule that Myc is obligate for tumor survival.

These observations strongly support the candidacy of Myc as a therapeutic target in many, most, or even (given its universal role in normal and tumor cell proliferation) all cancers. Although pharmacological inhibition of Myc currently remains a pipe dream such studies indicate that there are, indeed, common, essential and functionally non-degenerate cancer targets. Hopefully, advances in drug design and implementation will some day soon allow us to drug the undruggable and offer renewed therapeutic hope to cancer patients.

## References

[1] Bergers G, Hanahan D (2008). Modes of resistance to anti-angiogenic therapy. Nat Rev Cancer.

[2] Coussens LM, Fingleton B, Matrisian LM (2002). Matrix metalloproteinase inhibitors and cancer: trials and tribulations. Science.

[3] Fingleton B (2003). Matrix metalloproteinase inhibitors for cancer therapy:the current situation and future prospects. Expert Opin Ther Targets.

[4] Miller KD, Sweeney CJ, Sledge GW (2005). Can tumor angiogenesis be inhibited without resistance?. EXS.

[5] Eilers M, Eisenman RN (2008). Myc's broad reach. Genes Dev.

[6] Cole MD, Henriksson M (2006). 25 years of the c-Myc oncogene. Semin Cancer Biol.

[7] Oster SK, Ho CS, Soucie EL, Penn LZ (2002). The myc oncogene: MarvelouslY Complex. Adv Cancer Res.

[8] Menssen A, Hermeking H (2002). Characterization of the c-MYC-regulated transcriptome by SAGE: identification and analysis of c-MYC target genes. Proc Natl Acad Sci U S A.

[9] O'Connell BC, Cheung AF, Simkevich CP, Tam W, Ren X, Mateyak MK, Sedivy JM (2003). A large scale genetic analysis of c-Myc-regulated gene expression patterns. J Biol Chem.

[10] Watson JD, Oster SK, Shago M, Khosravi F, Penn LZ (2002). Identifying genes regulated in a Myc-dependent manner. J Biol Chem.

[11] Schuhmacher M, Kohlhuber F, Holzel M, Kaiser C, Burtscher H, Jarsch M, Bornkamm GW, Laux G, Polack A, Weidle UH (2001). The transcriptional program of a human B cell line in response to Myc. Nucleic Acids Res.

[12] Schuldiner O, Benvenisty N (2001). A DNA microarray screen for genes involved in c-MYC and N-MYC oncogenesis in human tumors. Oncogene.

[13] Guo QM, Malek RL, Kim S, Chiao C, He M, Ruffy M, Sanka K, Lee NH, Dang CV, Liu ET (2000). Identification of c-myc responsive genes using rat cDNA microarray. Cancer Res.

[14] Shiio Y, Donohoe S, Yi EC, Goodlett DR, Aebersold R, Eisenman RN (2002). Quantitative proteomic analysis of Myc oncoprotein function. EMBO J.

[15] Fernandez PC, Frank SR, Wang L, Schroeder M, Liu S, Greene J, Cocito A, Amati B (2003). Genomic targets of the human c-Myc protein. Genes Dev.

[16] Dang CV, O'Donnell KA, Zeller KI, Nguyen T, Osthus RC, Li F (2006). The c-Myc target gene network. Semin Cancer Biol.

[17] Meyer N, Penn LZ (2008). Reflecting on 25 years with MYC. Nat Rev Cancer.

[18] Davis AC, Wims M, Spotts GD, Hann SR, Bradley A (1993). A null c-myc mutation causes lethality before 10.5 days of gestation in homozygotes and reduced fertility in heterozygous female mice. Genes Dev.

[19] Mateyak MK, Obaya AJ, Adachi S, Sedivy JM (1997). Phenotypes of c-Myc-deficient rat fibroblasts isolated by targeted homologous recombination. Cell Growth Differ.

[20] Evan G, Littlewood T (1998). A matter of life and cell death. Science.

[21] Evan GI, Littlewood TD (1993). The role of c-myc in cell growth. Curr Opin Genet Dev.

[22] Harrington EA, Fanidi A, Evan GI (1994). Oncogenes and cell death. Curr Opin Genet Dev.

[23] Evan GI, Wyllie AH, Gilbert CS, Littlewood TD, Land H, Brooks M, Waters CM, Penn LZ, Hancock DC (1992). Induction of apoptosis in fibroblasts by c-myc protein. Cell.

[24] Hoffman B, Liebermann DA (2008). Apoptotic signaling by c-MYC. Oncogene.

[25] Murphy DJ, Junttila MR, Pouyet L, Karnezis A, Shchors K, Bui DA, Brown-Swigart L, Johnson L, Evan GI (2008). Distinct thresholds govern Myc's biological output in vivo. Cancer Cell.

[26] Lowe SW, Cepero E, Evan G (2004). Intrinsic tumour suppression. Nature.

[27] Littlewood TD, Hancock DC, Danielian PS, Parker MG, Evan GI (1995). A modified oestrogen receptor ligand-binding domain as an improved switch for the regulation of heterologous proteins. Nucleic Acids Res.

[28] Pelengaris S, Littlewood T, Khan M, Elia G, Evan G (1999). Reversible activation of c-Myc in skin: induction of a complex neoplastic phenotype by a single oncogenic lesion. Mol Cell.

[29] Pelengaris S, Khan M, Evan GI (2002). Suppression of Myc-induced apoptosis in beta cells exposes multiple oncogenic properties of Myc and triggers carcinogenic progression. Cell.

[30] Shachaf CM, Kopelman AM, Arvanitis C, Karlsson A, Beer S, Mandl S, Bachmann MH, Borowsky AD, Ruebner B, Cardiff RD (2004). MYC inactivation uncovers pluripotent differentiation and tumour dormancy in hepatocellular cancer. Nature.

[31] Podsypanina K, Politi K, Beverly LJ, Varmus HE (2008). Oncogene cooperation in tumor maintenance and tumor recurrence in mouse mammary tumors induced by Myc and mutant Kras. Proc Natl Acad Sci U S A.

[32] D'Cruz CM, Gunther EJ, Boxer RB, Hartman JL, Sintasath L, Moody SE, Cox JD, Ha SI, Belka GK, Golant A (2001). c-MYC induces mammary tumorigenesis by means of a preferred pathway involving spontaneous Kras2 mutations. Nat Med.

[33] Felsher DW, Bishop JM (1999). Reversible tumorigenesis by MYC in hematopoietic lineages. Mol Cell.

[34] Boxer RB, Jang JW, Sintasath L, Chodosh LA (2004). Lack of sustained regression of c-MYC-induced mammary adenocarcinomas following brief or prolonged MYC inactivation. Cancer Cell.

[35] Arvanitis C, Felsher DW (2005). Conditionally MYC: insights from novel transgenic models. Cancer Lett.

[36] Flores I, Murphy DJ, Swigart LB, Knies U, Evan GI (2004). Defining the temporal requirements for Myc in the progression and maintenance of skin neoplasia. Oncogene.

[37] Jain M, Arvanitis C, Chu K, Dewey W, Leonhardt E, Trinh M, Sundberg CD, Bishop JM, Felsher DW (2002). Sustained loss of a neoplastic phenotype by brief inactivation of MYC. Science.

[38] Dansen TB, Whitfield J, Rostker F, Brown-Swigart L, Evan GI (2006). Specific requirement for Bax, not Bak, in Myc-induced apoptosis and tumor suppression in vivo. J Biol Chem.

[39] Lawlor ER, Soucek L, Brown-Swigart L, Shchors K, Bialucha CU, Evan GI (2006). Reversible kinetic analysis of Myc targets in vivo provides novel insights into Myc-mediated tumorigenesis. Cancer Res.

[40] Shchors K, Shchors E, Rostker F, Lawlor ER, Brown-Swigart L, Evan GI (2006). The Myc-dependent angiogenic switch in tumors is mediated by interleukin 1beta. Genes Dev.

[41] Soucek L, Lawlor ER, Soto D, Shchors K, Swigart LB, Evan GI (2007). Mast cells are required for angiogenesis and macroscopic expansion of Myc-induced pancreatic islet tumors. Nat Med.

[42] Baudino TA, McKay C, Pendeville-Samain H, Nilsson JA, Maclean KH, White EL, Davis AC, Ihle JN, Cleveland JL (2002). c-Myc is essential for vasculogenesis and angiogenesis during development and tumor progression. Genes Dev.

[43] Soucek L, Jucker R, Panacchia L, Ricordy R, Tato F, Nasi S (2002). Omomyc, a potential Myc dominant negative, enhances Myc-induced apoptosis. Cancer Res.

[44] Soucek L, Helmer-Citterich M, Sacco A, Jucker R, Cesareni G, Nasi S (1998). Design and properties of a Myc derivative that efficiently homodimerizes. Oncogene.

[45] Savino M, Annibali D, Carucci N, Favuzzi E, Cole MD, Evan GI, Soucek L, Nasi S (2011). The action mechanism of the myc inhibitor termed omomyc may give clues on how to target myc for cancer therapy. PLoS One.

[46] Jackson EL, Willis N, Mercer K, Bronson RT, Crowley D, Montoya R, Jacks T, Tuveson DA (2001). Analysis of lung tumor initiation and progression using conditional expression of oncogenic K-ras. Genes Dev.

[47] Hanahan D (1985). Heritable formation of pancreatic beta-cell tumours in transgenic mice expressing recombinant insulin/simian virus 40 oncogenes. Nature.

[48] Soucek L, Whitfield J, Martins CP, Finch AJ, Murphy DJ, Sodir NM, Karnezis AN, Swigart LB, Nasi S, Evan GI (2008). Modelling Myc inhibition as a cancer therapy. Nature.

[49] Sodir NM, Swigart LB, Karnezis AN, Hanahan D, Evan GI, Soucek L (2011). Endogenous Myc maintains the tumor microenvironment. Genes Dev.

[50] Whitfield JR, Soucek L (2011). Tumor microenvironment: becoming sick of Myc. Cell Mol Life Sci.

